# Liquid biopsy monitoring uncovers acquired RAS-mediated resistance to cetuximab in a substantial proportion of patients with head and neck squamous cell carcinoma

**DOI:** 10.18632/oncotarget.8943

**Published:** 2016-04-22

**Authors:** Friederike Braig, Minna Voigtlaender, Aneta Schieferdecker, Chia-Jung Busch, Simon Laban, Tobias Grob, Malte Kriegs, Rainald Knecht, Carsten Bokemeyer, Mascha Binder

**Affiliations:** ^1^ Department of Oncology and Hematology with Sections Bone Marrow Transplant and Pneumology, Hubertus Wald Tumorzentrum / University Cancer Center Hamburg, University Medical Center Hamburg, Hamburg, Germany; ^2^ Department of Otorhinolaryngology, Head and Neck Cancer Center of The University Cancer Center Hamburg, University Medical Center Hamburg, Hamburg, Germany; ^3^ Department of Otorhinolaryngology and Head and Neck Surgery, Ulm University Medical Center, Ulm, Germany; ^4^ Department of Pathology, University Medical Center Hamburg, Hamburg, Germany; ^5^ Laboratory for Radiobiology and Experimental Radiooncology, Head and Neck Cancer Center of The University Cancer Center Hamburg, University Medical Center Hamburg, Hamburg, Germany

**Keywords:** head and neck squamous cell carcinoma (HNSCC), epidermal growth factor receptor (EGFR), RAS, resistance, cetuximab

## Abstract

Resistance to epidermal growth factor receptor (EGFR)-targeted therapy is insufficiently understood in head and neck squamous cell carcinoma (HNSCC), entailing the lack of predictive biomarkers.

Here, we studied resistance-mediating EGFR ectodomain and activating RAS mutations by next-generation sequencing (NGS) of cell lines and tumor tissue of cetuximab-naïve patients (46 cases, 12 cell lines), as well as liquid biopsies taken during and after cetuximab/platinum/5-fluorouracil treatment (20 cases). Tumors of cetuximab-naïve patients were unmutated, except for HRAS mutations in 4.3% of patients. Liquid biopsies revealed acquired KRAS, NRAS or HRAS mutations in more than one third of patients after cetuximab exposure. 46% of patients with on-treatment disease progression showed acquired RAS mutations, while no RAS mutations were found in the non-progressive subset of patients, indicating that acquisition of RAS mutant clones correlated significantly with clinical resistance (Chi square *p*=0.032). The emergence of mutations preceded clinical progression in half of the patients, with a maximum time from mutation detection to clinical progression of 16 weeks.

RAS mutations account for acquired resistance to EGFR-targeting in a substantial proportion of HNSCC patients, even though these tumors are rarely mutated at baseline. Liquid biopsies may be used for mutational monitoring to guide treatment decisions.

## INTRODUCTION

Head and neck squamous cell carcinomas (HNSCC) arising in the larynx, pharynx, oral cavity, paranasal sinuses and nasal cavity are among the most common types of cancers, accounting for almost 60,000 newly diagnosed cases and more than 10,000 estimated deaths per year in the United States alone [1]. The prognosis of HNSCC patients with locoregionally advanced tumors is poor as indicated by five year overall survival rates of 40-60% in recent clinical trials [2–6]. In recurrent and metastatic disease the mean overall survival does not exceed 11 months despite intensive treatment protocols [7, 8].

Cetuximab, a monoclonal antibody targeting the extracellular ligand binding domain of the epidermal growth factor receptor (EGFR), is approved for the treatment of locoregionally advanced HNSCC in combination with radiotherapy [9] and for the treatment of recurrent or metastatic disease in combination with platinum-based chemotherapy [7]. However, not all patients treated with cetuximab respond well to therapy due to primary or acquired resistance, limiting significantly the clinical benefit of this drug.

Still, the molecular mechanisms underlying clinical resistance to cetuximab in HNSCC have not yet been elucidated. In metastatic colorectal cancer (mCRC) resistance mechanisms are by far better understood and involve mutations in EGFR downstream signaling molecules such as RAS [10]. Constitutive RAS signaling is mediated by mutations that prevent GTP hydrolysis, thus locking RAS in a permanently active state, independent of EGFR engagement. For this reason, colon tumors harboring activating RAS mutations do not respond to EGFR targeting and mutational screening is therefore routinely used for patient selection prior to treatment [11, 12]. In HNSCC, however, primary RAS mutations are rather uncommon with only 4.6% of HRAS mutated tumors and their significance for this entity remains unclear [13, 14]. Just as primary resistance, acquired resistance to cetuximab represents a challenge in the treatment of both mCRC and HNSCC. A recent series of pivotal studies on mCRC suggested that acquired resistance to cetuximab may not only be mediated by selection of rare RAS mutated subclones (from predominantly RAS wildtype tumors) [15, 16] but also by acquisition of epitope-modifying EGFR mutations during cetuximab (or panitumumab) treatment [17–19]. In fact, the extracellular domain mutations R451C, S464L, G465R, K467T, I491M and S492R of the EGFR (all located in exon 12) were found in post-therapeutic tumor subclones or antibody-resistant cell lines by next-generation or sanger sequencing. These mutations abrogated antibody binding and, therefore, resulted in clinical resistance to cetuximab and/or panitumumab depending on their localization within the antibody epitopes [17, 19].

To investigate if these or related mechanisms may play a role in cetuximab resistance of HNSCC as well, we set out to scan the cetuximab-interacting ectodomain of the EGFR as well as KRAS/NRAS exons 2/3/4 and HRAS exons 2/3 for mutations in a cohort of 46 HNSCC patients by targeted next generation sequencing, 20 of these with available post-cetuximab circulating tumor DNA (ctDNA). We found that RAS mutations can be acquired in a substantial proportion of patients during cetuximab-based treatment and significantly correlate with disease progression. Future studies should quantitatively determine mutational loads that reliably predict the benefit - or lack thereof - from further cetuximab treatment in patients with acquired RAS mutations.

## RESULTS

### Patients and treatment

Of the 46 cetuximab-treated patients, 13 patients (28%) were in a curative and 33 patients (72%) were in a palliative treatment setting (Table 1). Although 19 of 46 patients (41%) had HNSCC of the oropharynx, HPV-positivity was rare with 5 of 46 patients (11%). The overall response rate (complete and partial responses) with cetuximab-based treatment was only 47% implying a high rate of treatment-resistant tumors in this cohort. About two thirds (13/20) of patients with liquid biopsies had progressive disease during combination therapy with Cis^−^ or carboplatin, 5-fluorouracil and cetuximab or cetuximab maintenance, respectively. Of the remaining seven patients without progressive disease, two patients refused cetuximab maintenance therapy, one patient died of pneumonia during combination therapy and one patient had severe bleeding complications requiring discontinuation of therapy. The median progression-free survival for patients in the liquid biopsy cohort was 4.9 months (95% CI 3.4-6.0), the median overall survival 5.2 months (95% CI 4.0-7.8).

**Table 1:** Patient and tumor characteristics with sequencing data of tumor samples

Pat.	Primary site	HPV-Status (p16/HPV-DNA)	Time of initial diagnosis		Time of relapse		Cetuximab treatment setting	Treatment combination	Origin oftumor sample	EGFR exon 12	KRASexon 2/3/4	NRASexon 2/3/4	HRASexon 2/3
			TNM	UICC	TNM	UICC							
1	Oro-/Hypopharynx	negative (−/−)	cT3 cN2c cM0	IV A	n.a.	n.a.	curative	RT + Cet	intial diagnosis	wt	wt/wt/wt	wt/wt/wt	**G13R/wt**
2	Hypopharynx/Larynx	negative (−/−)	cT4a cN2c cM0	IV A	n.a.	n.a.	curative	TPF; RT + Cet	intial diagnosis	wt	wt/wt/wt	wt/wt/wt	wt/wt
3	Oropharynx	negative (−/−)	pT2 pN2c cM1	IV C	n.a.	n.a.	palliative	CDDP, 5-FU, Cet	intial diagnosis	wt	wt/wt/wt	wt/wt/wt	wt/wt
4	Oral Cavity	negative (−/−)	cT4a cN2c cM0	IV A	n.a.	n.a.	curative	TPF; RT + Cet	intial diagnosis	wt	wt/wt/wt	wt/wt/wt	wt/wt
5	Oropharynx	negative (−/−)	cT2 cN2b cM0	IV A	n.a.	n.a.	curative	TPF; RT + Cet	relapse	wt	wt/wt/wt	wt/wt/wt	wt/wt
6	Oral Cavity	negative (−/−)	pT2 pN2b cM0	IV A	rcT2 cN0 cM0	II	palliative	CDDP, 5-FU, Cet	relapse	wt	wt/wt/wt	wt/wt/wt	wt/wt
7	Oropharynx	negative (−/−)	cT4a cN2c cM1	IV C	n.a.	n.a.	palliative	CDDP, 5-FU, Cet	intial diagnosis	wt	wt/wt/wt	wt/wt/wt	wt/wt
8	Oropharynx	positive (+/+)	cT3 cN2b cM0	IV A	n.a.	n.a.	curative	RT + Cet	intial diagnosis	wt	wt/wt/wt	wt/wt/wt	wt/wt
9	Hypopharynx	negative (−/+)	cT2 cN2c cM0	IV A	n.a.	n.a.	curative	TPF; RT + Cet	intial diagnosis	wt	wt/wt/wt	wt/wt/wt	wt/wt
10	Larynx	negative (−/−)	cT2 cN3 cM1	IV C	n.a.	n.a.	palliative	CDDP, 5-FU, Cet	intial diagnosis	wt	wt/wt/wt	wt/wt/wt	wt/wt
11	Oropharynx	positive (+/+)	pT1 pN2b cM0	IV A	rcT0 cN0 cM1	IV C	palliative	CDDP, 5-FU, Cet	intial diagnosis	wt	wt/wt/wt	wt/wt/wt	n.e./n.e.
12	Oropharynx	negative (−/−)	cT4a cN1 cM0	IV A	rcT2 cN1 cM0	III	palliative	Carbo, Taxol, Cet	relapse	wt	wt/wt/wt	wt/wt/wt	n.e./wt
13	Hypopharynx	negative (−/−)	cT4a cN2c cM0	IV A	n.a.	n.a.	curative	TPF; RT + Cet	relapse	wt	wt/wt/wt	wt/wt/wt	wt/wt
14	Larynx	negative (−/−)	cT2 cN0 cM0	II	n.a.	n.a.	curative	TPF; RT + Cet	intial diagnosis	wt	wt/wt/wt	wt/wt/wt	wt/wt
15	Oral Cavity	negative (−/−)	cT4a cN2c cM0	IV A	n.a.	n.a.	curative	RT + Cet	intial diagnosis	wt	wt/wt/wt	wt/wt/wt	wt/wt
16	Oropharynx	negative (−/−)	cT4a cN2b cM0	IV A	rcT4a cN2b cM0	IV A	palliative	Carbo, Taxol, Cet	intial diagnosis	wt	wt/wt/wt	wt/wt/wt	wt/wt
17	Paranasal Sinus	negative (−/−)	pT4a pN0 cM0	IV A	rcT4a cN2c cM0	IV A	palliative	CDDP, 5-FU, Cet	intial diagnosis	wt	wt/wt/wt	wt/wt/wt	wt/wt
18	Oro-/Hypopharynx	negative (−/−)	pT3 pN1 cM0	III	rcT3 cN0 cM0	III	palliative	RT + Cet	relapse	wt	wt/wt/wt	wt/wt/wt	wt/wt
19	Oropharynx	negative (−/−)	cTx cNx cM0	n.a.	rcT4a cN2b M1	IV C	palliative	CDDP, 5-FU, Cet	intial diagnosis	wt	wt/wt/wt	wt/wt/wt	wt/wt
20	Paranasal Sinus	negative (−/−)	cT4a cN2c cM0	IV A	rcT4a cN0 cM0	IV A	palliative	Gem, Vino, Cet	intial diagnosis	wt	wt/wt/wt	wt/wt/wt	wt/wt
21	Oral Cavity	negative (−/−)	cT4a cN2c cM0	IV A	rcT3 cN0 cM1	IV C	palliative	CDDP, 5-FU, Cet	intial diagnosis	wt	wt/wt/wt	wt/wt/wt	wt/wt
22	Hypopharynx	negative (−/−)	cT3 cN2b cM0	IV A	n.a.	n.a.	curative	TPF; RT + Cet	intial diagnosis	wt	wt/wt/wt	wt/wt/wt	wt/wt
23	Oral Cavity	negative (−/−)	cT4a cN2c cM1	IV C	n.a.	n.a.	palliative	Carbo, Taxol, Cet	intial diagnosis	wt	wt/wt/wt	wt/wt/wt	n.e./n.e.
24	Oral Cavity	negative (−/−)	cT3 cN2c cM0	IV A	n.a.	n.a.	curative	RT + Cet	intial diagnosis	wt	wt/wt/wt	wt/wt/wt	wt/wt
25	Orophaynx	negative (−/−)	cT4a cN2c cM0	IV A	n.a.	n.a.	curative	TPF; RT + Cet	intial diagnosis	wt	wt/wt/wt	wt/wt/wt	wt/wt
26	Oropharynx	positive (+/+)	cT4a cN2c cM0	IV A	n.a.	n.a.	curative	RT + Cet	intial diagnosis	wt	wt/wt/wt	wt/wt/wt	wt/wt
27	Hypopharynx	negative (−/−)	pT2 pN1 cM0	III	rcT4a cN0 cM0	IV A	palliative	CDDP, 5-FU, Cet	relapse	wt	wt/wt/wt	wt/wt/wt	wt/wt
28	Larynx	negative (−/−)	cT2 cN0 cM0	II	cT2 cN1 cM0	III	palliative	CDDP, 5-FU, Cet	relapse	wt	wt/wt/wt	wt/wt/wt	wt/wt
29	Hypopharynx	negative (−/−)	cT4a cN3 cM1	IV C	n.a.	n.a.	palliative	CDDP, 5-FU, Cet	intial diagnosis	wt	wt/wt/wt	wt/wt/wt	wt/wt
30	Oral Cavity	negative (+/−)	pT2 pN0 cM0	II	rcT3 cN0 cM1	IV C	palliative	CDDP, 5-FU, Cet	intial diagnosis	wt	wt/wt/wt	wt/wt/wt	**G13R/wt**
31	Oropharynx	negative (−/−)	pT2 pN2b cM0	IV A	rcTx cN1 cM1	IV C	palliative	CDDP, 5-FU, Cet	relapse	wt	wt/wt/wt	wt/wt/wt	wt/wt
32	Oral Cavity	negative (−/−)	cT4b cN3 cM1	IV B	n.a.	n.a.	palliative	CDDP, 5-FU, Cet	intial diagnosis	wt	wt/wt/wt	wt/wt/wt	wt/wt
33	Larynx	negative (−/−)	pT2 pN2b cM0	IV A	rcT3 cN0 cM0	III	palliative	CDDP, 5-FU, Cet	relapse	wt	wt/wt/wt	wt/wt/wt	wt/wt
34	Oropharynx	positive (+/+)	pT3 pN2c cM0	IV A	rcTx cNx cM1	IV C	palliative	CDDP, 5-FU, Cet	relapse	wt	wt/wt/wt	wt/wt/wt	wt/wt
35	Hypopharynx	negative (−/+)	cT3 cN2b cM0	IV A	rcTx cN3 cM1	IV C	palliative	CDDP, 5-FU, Cet	intial diagnosis	wt	wt/wt/wt	wt/wt/wt	wt/wt
36	Oral Cavity	negative (−/−)	pT2 pN3 cM0	IV B	rcTx cN1 cM1	IV C	palliative	CDDP, 5-FU, Cet	intial diagnosis	wt	wt/wt/wt	wt/wt/wt	wt/wt
37	Oral Cavity	negative (−/−)	pT4a pN0 cM0	IV A	rcT3 cN1 cM0	III	palliative	CDDP, 5-FU, Cet	intial diagnosis	wt	wt/wt/wt	wt/wt/wt	wt/wt
38	Oral Cavity	negative (−/−)	cT4a cN1 cM0	IV A	rcT3 cN2 cM1	IV C	palliative	CDDP, 5-FU, Cet	intial diagnosis	wt	wt/wt/wt	wt/wt/wt	wt/wt
39	Oropharynx	positive (+/+)	cT3 cN0 cM0	III	cT2 cN1 cM0	III	palliative	CDDP, 5-FU, Cet	relapse	wt	wt/wt/wt	wt/wt/wt	wt/wt
40	Hypopharynx	negative (−/−)	pT2 pN2c cM0	IV A	rcT2 cN2c cM0	IV A	palliative	CDDP, 5-FU, Cet	relapse	wt	wt/wt/wt	wt/wt/wt	wt/wt
41	Oral Cavity	negative (−/−)	cT4b cN2c cM1	IV C	n.a.	n.a.	palliative	CDDP, 5-FU, Cet	intial diagnosis	wt	wt/wt/wt	wt/wt/wt	n.e./wt
42	Oropharynx	negative (−/−)	pT2 pN1 cM0	III	rcT0 cN0 cM1	IV C	palliative	CDDP, 5-FU, Cet	intial diagnosis	wt	wt/wt/wt	wt/wt/wt	wt/wt
43	Hypopharynx	negative (−/−)	cT2 cN2b cM1	IV C	n.a.	n.a.	palliative	CDDP, 5-FU, Cet	intial diagnosis	wt	wt/wt/wt	wt/wt/wt	wt/wt
44	Oropharynx	negative (−/−)	pT1 pN2b cM0	IV A	rcT2 cN0 cM1	IV C	palliative	CDDP, 5-FU, Cet	intial diagnosis	wt	wt/wt/wt	wt/wt/wt	wt/wt
45	Oropharynx	negative (−/−)	cT4a cN2c cM0	IV A	rcT0 cN2c cM1	IV C	palliative	CDDP, 5-FU, Cet	relapse	wt	wt/wt/wt	wt/wt/wt	wt/wt
46	Oropharynx	negative (−/−)	cT1 cN3 cM0	IV B	cT4 cN0 cM0	IV A	palliative	CDDP, 5-FU, Cet	relapse	wt	wt/wt/wt	wt/wt/wt	wt/wt

Abbreviations:*HPV* human papilloma virus; *n.a.* not applicable; *RT* radiotherapy; *Cet* Cetuximab; *TPF* Docetaxel/Cisplatin/5-Fluoruracil; *CDDP* 5-FU Cis-(or Carbo)platin/5-Fluoruracil; *Carbo, Taxol* Carboplatin/Paclitaxel; *Gem*, Vino Gemcitabine/Vinorelbine; *wt* wildtype; *n.e.* not evaluable

### NGS of the cetuximab-interacting EGFR ectodomain and RAS at baseline and in HNSCC cell lines

**Figure 1:**
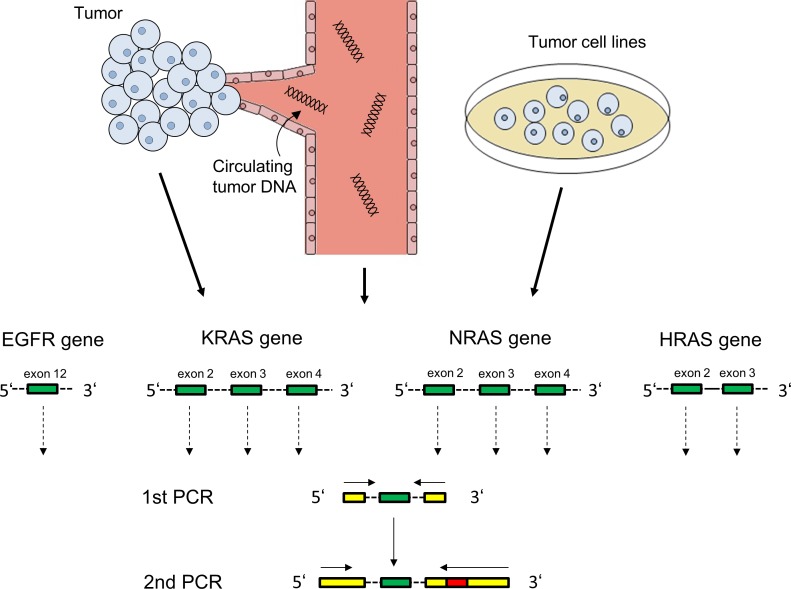
PCR amplification of EGFR and RAS exons for Illumina targeted next generation sequencing EGFR exon 12, KRAS/NRAS exons 2/3/4 and HRAS exons 2/3 (green) were amplified from tumor tissue of 46 patients, post-cetuximab circulating tumor DNA of 20 patients and from 12 squamous carcinoma cell lines. Illumina-specific sequences for hybridization and sequencing (yellow) as well as patient-specific barcodes (red) were attached in a second PCR step.

We sought to find out i) if tumor subclones expressing a mutated EGFR ectodomain or activating RAS mutations exist in HNSCC tumors before cetuximab-based treatment and ii) if such subclones emerge or expand under the selective pressure of EGFR-directed antibody treatment in this disease. We used NGS to screen EGFR exon 12, KRAS/NRAS exons 2/3/4 and HRAS exons 2/3 with a mean number of > 20,000 reads per exon, ensuring that even rare mutant subclones would be detected (targeted NGS approach schematically shown in Figure 1).

None of the tumor tissue samples of all 46 patients showed evidence of mutations in the cetuximab-interacting EGFR ectodomain or KRAS/NRAS. In line with previous reports, activating HRAS mutations were found in primary tumor samples of two patients (4.3%) with one clonal (patient no. 1) and one subclonal mutation (patient no. 30), (Table 1).

All 12 HNSCC cell lines that derived from EGFR antibody-naïve patients were unmutated for EGFR, KRAS/NRAS and HRAS (Table 2).

**Table 2:** Characteristics and sequencing data of squamous cell carcinoma cell lines

Cellline	Origin	HPV	EGFRexon 12	KRASexon 2/3/4	NRASexon 2/3/4	HRAS exon 2/3	Reference
UT-SCC-5	Tongue	negative	wt	wt/wt/wt	wt/wt/wt	wt/wt	Lin et al. [25]
UT-SCC-8	Supraglotticlarynx	negative	wt	wt/wt/wt	wt/wt/wt	wt/wt	Lin et al. [25]
UT-SCC-14	Tongue	negative	wt	wt/wt/wt	wt/wt/wt	wt/wt	Lin et al. [25]
UT-SCC-15	Tongue	negative	wt	wt/wt/wt	wt/wt/wt	wt/wt	Lin et al. [25]
UT-SCC-29	Glotticlarynx	negative	wt	wt/wt/wt	wt/wt/wt	wt/wt	Lin et al. [25]
UT-SCC-42A	Supraglottis	negative	wt	wt/wt/wt	wt/wt/wt	wt/wt	Lin et al. [25]
UT-SCC-60A	Tonsil	negative	wt	wt/wt/wt	wt/wt/wt	wt/wt	Lange et al. [22]
Cal33	Tongue	negative	wt	wt/wt/wt	wt/wt/wt	wt/wt	Soffar et al. [24]
HSC-4	Tongue	negative	wt	wt/wt/wt	wt/wt/wt	wt/wt	Lin et al. [25]
FaDu	Hypopharynx	negative	wt	wt/wt/wt	wt/wt/wt	wt/wt	Eicheler et al. [26]
SAS	Tongue	negative	wt	wt/wt/wt	wt/wt/wt	wt/wt	Soffar et al. [24]
SAT	Oral cavity	negative	wt	wt/wt/wt	wt/wt/wt	wt/wt	Nii et al. [23]

Abbreviation: *wt wildtype*

### NGS of the cetuximab-interacting EGFR ectodomain and RAS after cetuximab treatment

**Figure 2:**
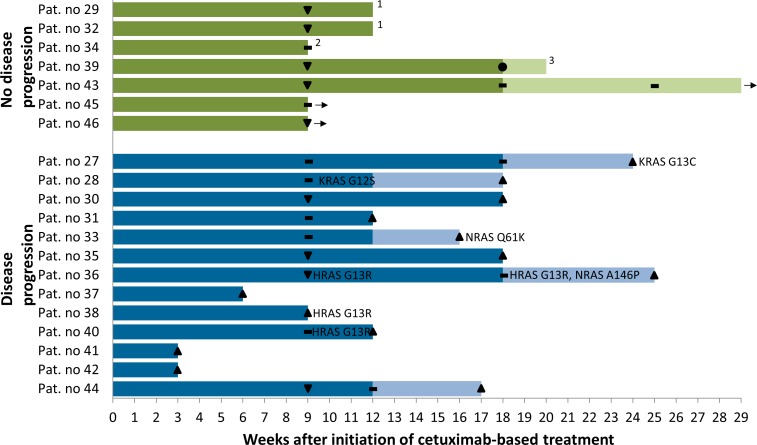
Swimmer plot illustrating treatment, responses and acquired mutations in liquid biopsy cohort of 20 HNSCC patients treated with cetuximab plus chemotherapy Weeks of combination therapy with cis^−^ or carboplatin, 5-fluorouracil and cetuximab are shown in dark colors, weeks of cetuximab maintenance in light colors. • Complete response, ▼ partial response, — stable disease, ▲ progressive disease. Activating RAS mutations are mapped at the time of their first appearance. ^1^Patients refused further treatment. ^2^Patient died of pneumonia. ^3^Therapy was stopped because of bleeding complications. → Ongoing treatment.

In 20 patients we obtained peripheral blood for ctDNA analysis during and after combination therapy with Cis^−^ or carboplatin, 5-fluorouracil and cetuximab +/− cetuximab maintenance (liquid biopsy). Overall, about one third of patients acquired activating RAS mutations in the course of cetuximab-based treatment (KRAS: G12S, G13C; NRAS: Q61K, A146P; HRAS: G13R), while no EGFR ectodomain mutations were recorded (Figure 2). The emergence of activating RAS clones correlated significantly with disease progression in this cohort (Chi-square, *P* = 0.032). While six of 13 patients (46%) with progressive disease during cetuximab-based treatment showed evidence of acquired activating RAS mutations, none of the seven responsive patients (0%) were mutated for any of the RAS genes at any time point (Figure 2). Some of these mutations appeared early during treatment (earliest detection nine weeks after initiation of cetuximab-based treatment) and preceded clinical progression in half of the patients with the maximum time from mutation detection to clinical progression being 16 weeks in our cohort (Figure 2).

## DISCUSSION

Cetuximab-based treatment is only effective in a subset of patients with HNSCC [7]. However, little is known so far about the molecular mechanisms underlying clinical resistance and we currently lack appropriate biomarkers that could help in identifying patient subsets that are either likely or unlikely to derive benefit from this EGFR-targeted therapy or from prolonged antibody treatment in a cetuximab maintenance setting.

In this study we focused on potential modifications of the EGFR ectodomain that may interfere with antibody binding and activating mutations of RAS, which are known to confer resistance in metastatic colorectal cancer [10, 19]. While HNSCC tumors are largely negative for RAS mutations at diagnosis [14, 20] and EGFR ectodomain mutations have not been detected by conventional sequencing to date, we reasoned that potential resistance-mediating mutations could be present in rare tumor subclones before treatment (undetectable by conventional sequencing) and would subsequently be amplified under the selective pressure of EGFR-targeted antibody treatment. To be able to detect even minor subclones in a background of cells with unmutated EGFR and RAS, we used state-of-the-art targeted NGS technology for highly sensitive and specific identification of mutations in a heterogeneous tumor [21]. By comparing pre- and post-cetuximab genetic material we aimed at uncovering both primary and acquired resistance-mediating mutations. Utilizing a sequencing depth that would uncover even rare clones, none of the 46 patients included in this study showed evidence for mutations in EGFR exon 12 or KRAS/NRAS exons 2/3/4 at baseline, while two cases were HRAS mutated. About one third of cases acquired RAS mutations in the course of treatment and, interestingly, all of these cases showed progressive disease while receiving the EGFR antibody. This significant correlation suggests for the first time that activating RAS mutations represent a clinically relevant mechanism of acquired resistance in patients treated with cetuximab.

Two major limitations of this study need to be discussed: First, this study does not formally rule out the (unlikely) possibility that the platinum/5-FU treatment (and not the EGFR-targeted antibody) may induce activating RAS mutations in the HNSCC setting. In the colon cancer setting, however, there is no evidence for the induction of activating RAS mutations by chemotherapy, while there is persuasive evidence for their induction by EGFR-targeting antibodies [15]. Since patients treated with either platinum/5-FU or cetuximab alone are rare, this question is very hard to address. The second limitation of our study refers to the fact that baseline mutational profiling was performed on primary diagnosis tumor tissue (instead of tumor tissue at recurrence) in 7/20 patients and baseline liquid biopsies were not performed. Therefore, we cannot rule out acquisition of mutations between primary diagnosis and recurrence in these seven patients. Given the overall very low RAS mutational frequency in EGFR antibody-naïve patients this point may, however, be of minor clinical relevance.

Taken together, our data suggests that i) RAS mutant subclones can only be found in a minority of HNSCC tumor samples at baseline, but emerge in a substantial proportion of patients during cetuximab treatment, ii) these mutant subclones correlate significantly with disease progression, and iii) may be detectable with state-of-the-art sequencing technology before clinical resistance occurs. Prospectively, determination of such clones may help to tailor anti-EGFR strategies warranting an evaluation in larger prospective clinical trials. More specifically, mutational loads should be defined that reliably predict a lack of response to cetuximab.

## METHODS - PATIENTS

Between October 2012 and January 2016, the Database of the Clinical Cancer Registry of the University Cancer Center Hamburg was screened for HNSCC patients with cetuximab-based treatment. Informed consent was obtained from a total of 46 patients for the use of their diagnostic material (tumor tissue and - in 20 cases - peripheral blood after initiation of cetuximab treatment) as approved by the institutional review board. HPV-Status was part of the routine diagnostic work-up and included a PCR for HPV-DNA and p16 immunohistochemistry. Patients were considered HPV-positive, if both HPV-DNA of a high-risk HPV type and overexpression of p16 were present. All other combinations were considered HPV-negative. All tumor samples were validated by a pathologist.

All 20 patients with available post-cetuximab peripheral blood samples were treated with a combination of cetuximab weekly (400 mg/m^2^ as a loading dose, followed by 250 mg/m^2^) and a chemotherapy regimen of cisplatin (100 mg/m^2^ on day 1) or carboplatin (AUC 5 on day 1) plus 5-fluorouracil (1000 mg/m^2^ on days 1-4) every three weeks for a maximum of six courses. Subject to their consent, combination therapy was followed by weekly cetuximab maintenance in patients without progressive disease. Peripheral blood samples for isolation of ctDNA were taken at interim staging after three courses of combination therapy and after completion of combination therapy / maintenance (or at progression if applicable).

## MATERIALS AND METHODS

### Cell lines and cell culture

All 12 squamos cell carcinoma cell lines derived from patients with head and neck tumors [22–25] were cultivated in Dulbecco's Modified Eagle's Medium (Gibco/Life Technologies, Carlsbad, USA) containing 10% fetal bovine serum (Merck & Co., Inc., Kenilworth, USA), 4 mM glutamine (Gibco/Life Technologies) and 1% Penicillin Streptomycin (Gibco/Life Technologies) and were identified using a short tandem repeat multiplex assay (Applied Biosystems/Life Technologies). UT-SCC cell lines 5, 8, 14, 15, 29, 42A and 60A were kindly provided by R. Grenman, University of Turku, Finland. The HNSCC p53-negative subline of FaDu (hypopharynx) was obtained from W. Eicheler, University of Dresden, Germany [26], and all other cell lines were kindly provided by M. Baumann, University of Dresden, Germany.

### Preparation of genomic DNA from tumor tissue and HNSCC cell lines

Formalin fixed paraffin embedded tumor tissue was deparaffinized by xylene and ethanol. After digestion with proteinase K at 56°C overnight, genomic DNA was isolated with the QIAamp DNA Micro Kit (Qiagen, Hilden, Germany). Genomic DNA from EGFR positive cell lines was extracted using NucleoSpin Tissue XS kit (Macherey-Nagel, Düren, Germany). Quantity and quality of DNA was evaluated using a Nanodrop spectrophotometer ND-1000 (Thermo Fisher Scientific Inc., Wilmington, USA).

### Isolation of circulating tumor DNA (ctDNA) from blood

Blood samples were centrifuged at 1200 x g for 10 min within two hours after blood draw. ctDNA was isolated from the serum using the QIAamp Circulating Nucleic Acid Kit (Qiagen).

### PCR amplification of EGFR and RAS exons for Illumina targeted next generation sequencing

In two consecutive PCR reactions, EGFR exon 12, KRAS/NRAS exons 2/3/4 and HRAS exons 2/3 were amplified from tumor or ctDNA and adapters for next-generation sequencing (NGS) were attached (schematically shown in Figure 1). The primers for the first reaction annealed with exon-flanking intron regions (dotted lines) and contained Illumina-compatible adapters (yellow) for later hybridization of amplicons to the Illumina flow cell and for sequencing primer annealing. A second PCR reaction was performed to extend the Illumina adapter sequences and to add a 6 or 7 nucleotide barcode (red) allowing to match each sequence during data analysis to a certain patient / cell line and time point. All primers are shown in Supplementary Table S1.

The PCR was performed using Phusion HS II (Thermo Fisher Scientific Inc.). Amplicons were purified after agarose gel electrophoresis using the NucleoSpin^®^ Gel and PCR Clean-up kit (Macherey-Nagel).

### Illumina sequencing and data analysis

All amplicons were sequenced with a 500-cycle single indexed (8 nucleotides) paired-end run on a MiSeq (Illumina, San Diego, USA). Overlapping paired reads were merged using the software FLASH (v1.2.6) [27]. The format of the merged reads was subsequently converted to FASTA while non-overlapping reads were excluded from further analysis. Usearch (v6.0.307) [28] was employed to dereplicate and cluster the merged reads. Sequences observed less than 30 times were discarded and the remaining sequences were clustered according to their similarity with reference EGFR and RAS exon sequences (Supplementary Table S2). For each cluster of similar sequences, MAFFT (v7.045b) [29] was used to calculate a multiple sequence alignment.

### Statistics

IBM^®^ SPSS^®^ version 22 (IBM, New York, USA) was used for statistical analysis. Contingency tables were calculated and compared using the Pearson Chi-square test. A *p*-value < 0.05 was considered significant.

## SUPPLEMENTARY MATERIAL AND TABLES


