# Supplementary material for: Liquid biopsy monitoring uncovers acquired RAS-mediated resistance to cetuximab in a substantial proportion of patients with head and neck squamous cell carcinoma
Source: Oncotarget. 2016 Apr 22;7(28):42988–95. doi: 10.18632/oncotarget.8943 (PMC5190002; doi:10.18632/oncotarget.8943)
Supplement: Supplementary file 1 [file oncotarget-07-42988-s001.pdf]

Liquid biopsy monitoring uncovers acquired RAS-mediated resistance to cetuximab in a substantial proportion of patients with head and neck squamous cell carcinoma

Supplementary Material

Supplementary Table 1: Illumina-sequencing primers.*

PCR	Primer name	Primer sequence 5’-3’
1st PCR	Seq_Exon 12_I_fw	ACACTCTTTCCCTACACGACGCTCTTCCGATCTtacattgtttttataatttttcaccac
	Seq_Exon 12_II_fw	ACACTCTTTCCCTACACGACGCTCTTCCGATCTcgctccctcaaggagataag
	Seq_KRAS_Exon 2_fw	ACACTCTTTCCCTACACGACGCTCTTCCGATCTgcctgctgaaaatgactgaa
	Seq_KRAS_Exon 3_fw	ACACTCTTTCCCTACACGACGCTCTTCCGATCTccagactgtgtttctcccttc
	Seq_KRAS_Exon 4_fw	ACACTCTTTCCCTACACGACGCTCTTCCGATCTggactctgaagatgtacctatgg
	Seq_NRAS_Exon 2_fw	ACACTCTTTCCCTACACGACGCTCTTCCGATCTgttcttgctggtgtgaaatgac
	Seq_NRAS_Exon 3_fw	ACACTCTTTCCCTACACGACGCTCTTCCGATCTattgaacttccctccctccc
	Seq_NRAS_Exon 4_fw	ACACTCTTTCCCTACACGACGCTCTTCCGATCTttcccgtttttagGGAGCAGA
	Seq_HRAS_Exon 2_fw	ACACTCTTTCCCTACACGACGCTCTTCCGATCTggcaggagaccctgtagga
	Seq_HRAS_Exon 3_fw	ACACTCTTTCCCTACACGACGCTCTTCCGATCTctgagccctgtcctcctg
	Seq_Exon 12_I_rv	TGACTGGAGTTCAGACGTGTGCTCTTCCGATCTtggttttctgaccggaggt
	Seq_Exon 12_II_rv	TGACTGGAGTTCAGACGTGTGCTCTTCCGATCTgacccattagaaccaactcca
	Seq_KRAS_Exon 2_rv	TGACTGGAGTTCAGACGTGTGCTCTTCCGATCTagaatggtcctgcaccagtaa
	Seq_KRAS_Exon 3_rv	TGACTGGAGTTCAGACGTGTGCTCTTCCGATCTtccctcattgcactgtactcc
	Seq_KRAS_Exon 4_rv	TGACTGGAGTTCAGACGTGTGCTCTTCCGATCTtcagtgttacttacctgtcttgt
	Seq_NRAS_Exon 2_rv	TGACTGGAGTTCAGACGTGTGCTCTTCCGATCTaagtggttctggattagctgga
	Seq_NRAS_Exon 3_rv	TGACTGGAGTTCAGACGTGTGCTCTTCCGATCTttggtctctcatggcactgt
	Seq_NRAS_Exon 4_rv	TGACTGGAGTTCAGACGTGTGCTCTTCCGATCTtgcaaactcttgcacaaatgc
	Seq_HRAS_Exon 2_rv	TGACTGGAGTTCAGACGTGTGCTCTTCCGATCTagccctatcctggctgtgt
	Seq_HRAS_Exon 3_rv	TGACTGGAGTTCAGACGTGTGCTCTTCCGATCTcaaacacacacaggaagccc
2nd PCR	Linker_Seq_fw	AATGATACGGCGACCACCGAGATCTACACTCTTTCCCTACACGAC
	Linker_Bar_Seq_rv	CAAGCAGAAGACGGCATACGAGAT(N_{6-7})GTGACTGGAGTTCAGACGTGTG

* *Capital letters* NGS specific sequences; *lower case letters* gen-specific sequences

Supplementary Table 2: EGFR and RAS reference exon sequences.

Exon name	Reference sequences 5’-3’
EGFR exon 12	TGGTCAGTTTTCTCTTGCAGTCGTCAGCCTGAACATAACAT<br>CCTTGGGATTACGCTCCCTCAAGGAGATAAGTGATGGAGA<br>TGTGATAATTTCAGGAAACAAAAATTTGTGCTATGCAAATAC<br>AATAAACTGGAAAAAACTGTTTGGGACCTCCGGTCAGAAAA<br>CCAAAATTATAAGCAACAGAGGTGAAAACAGCTGCA
KRAS exon 2	GCCTGCTGAAAATGACTGAATATAAACTTGTGGTAGTTGGA<br>GCTGGTGGCGTAGGCAAGAGTGCCTTGACGATACAGCTAA<br>TTCAGAATCATTTTGTGGACGAATATGATCCAACAATAGAG
KRAS exon 3	GATTCCTACAGGAAGCAAGTAGTAATTGATGGAGAAACCT<br>GTCTCTTGGATATTCTCGACACAGCAGGTCAAGAGGAGTA<br>CAGTGCAATGAGGGACCAGTACATGAGGACTGGGGAGGG<br>CTTTCTTTGTGTATTTGCCATAAATAATACTAAATCATTTGA<br>AGATATTCACCATTATAG
KRAS exon 4	GGACTCTGAAGATGTACCTATGGTCCTAGTAGGAAATAAAT<br>GTGATTTGCCTTCTAGAACAGTAGACACAAAACAGGCTCAG<br>GACTTAGCAAGAAGTTATGGAATTCCTTTTATTGAAACATCA<br>GCAAAGACAAGACAG
NRAS exon 2	GTTCTTGCTGGTGTGAAATGACTGAGTACAAACTGGTGGT<br>GGTTGGAGCAGGTGGTGTTGGGAAAAGCGCACTGACAATC<br>CAGCTAATCCAGAACCACTT
NRAS exon 3	GATTCTTACAGAAAACAAGTGGTTATAGATGGTGAAACCTG<br>TTTGTTGGACATACTGGATACAGCTGGACAAGAAGAGTACA<br>GTGCCATGAGAGACCAA
NRAS exon 4	GGAGCAGATTAAGCGAGTAAAAGACTCGGATGATGTACCT<br>ATGGTGCTAGTGGGAAACAAGTGTGATTTGCCAACAAGGA<br>CAGTTGATACAAAACAAGCCCACGAACTGGCCAAGAGTTA<br>CGGGATTCCATTCATTGAAACCTCAGCCAAGACCAGACAG
HRAS exon 2	GGCAGGAGACCCTGTAGGAGGACCCCGGGCCGCAGGCC<br>CCTGAGGAGCGATGACGGAATATAAGCTGGTGGTGGTGG<br>GCGCCGGCGGTGTGGGCAAGAGTGCGCTGACCATCCAGC<br>TGATCCAGAACCATTTTGTGGACGAATACGACCCCACTATA<br>GAG
HRAS exon 3	GATTCCTACCGGAAGCAGGTGGTCATTGATGGGGAGACGT<br>GCCTGTTGGACATCCTGGATACCGCCGGCCAGGAGGAGT<br>ACAGCGCCATGCGGGACCAGTACATGCGCACCGGGGAGG<br>GCTTCCTGTGTGTGTTTG
